# Hypogammaglobulinémie acquise associée au thymome: le syndrome de Good

**DOI:** 10.11604/pamj.2017.28.253.11352

**Published:** 2017-11-22

**Authors:** Samira Aouadi, Najla Ghrairi, Emna Braham, Manel Kaabi, Sonia Maâlej, Leila Douik Elgharbi

**Affiliations:** 1 Service de Pneumologie D, Université de Tunis El Manar, Faculté de Médecine de Tunis, Hôpital Abderrahmen Mami, Ariana, Tunisie; 2 Laboratoire d’Immunologie Université de Tunis El Manar, Faculté de Médecine de Tunis, Hôpital Abderrahmen Mami, Ariana, Tunisie; 3 Laboratoire d’Anatomie Pathologique, Université de Tunis El Manar, Faculté de Médecine de Tunis, Hôpital Abderrahmen Mami, Ariana, Tunisie

**Keywords:** Thymome, hypogammaglobulinémie, syndrome de Good, Thymoma, hypogammaglobulinemia, Good syndrome

## Abstract

Le syndrome de Good (SG) est défini par l'association d'un thymome et d'un déficit immunitaire. Il se complique souvent d’infections bactériennes broncho-pulmonaires et rhino-sinusiennes. Cette entité ne représente que 5% des syndromes para-thymiques. Ces infections respiratoires récurrentes peuvent être à l’origine de dilatation des bronches associée au syndrome de Good. Nous rapportons l’observation d’une femme âgée de 52 ans, hospitalisée pour une pneumopathie infectieuse trainante. La tomodensitométrie thoracique a permis de mettre en évidence des dilatations des bronches associées à un thymome confirmé sur pièce opératoire. La découverte d’une hypogammaglobulinémie a permis de porter le diagnostic de syndrome de Good.

## Abstract (en)

Good syndrome (GS) is defined as the association between thymoma and immune deficiency. It is often complicated by broncho-pulmonary bacterial infections and rhinosinusitis. This disease accounts for only 5% of all parathymic syndromes. These recurrent respiratory infections can cause bronchiectasis associated with Good syndrome. We report the case of a 52-year old woman hospitalized for non resolutive infectious pneumonitis. Chest CT scan showed bronchiectasis associated with thymoma confirmed by biopsy. The discovery of hypogammaglobulinemia allowed the diagnosis of Good syndrome.

## Introduction

Le syndrome de Good (SG) est défini par l´association d´un thymome et d´un déficit immunitaire. Il a été rapporté pour la première fois il y a près de 50 ans par Robert Good [1]. Cette entité ne représente que 5 % des syndromes para-thymiques [2]. Ce syndrome est à l’origine d’une dysrégulation de la réponse immunitaire aboutissant à la fois à un déficit immunitaire et à une auto-immunité. Il se complique souvent d’infections bactériennes broncho-pulmonaires et rhino-sinusiennes [3]. Ces infections respiratoires récurrentes peuvent être à l’origine de dilatation des bronches associée au syndrome de Good [4].

## Patient et observation

**Figure 1:**
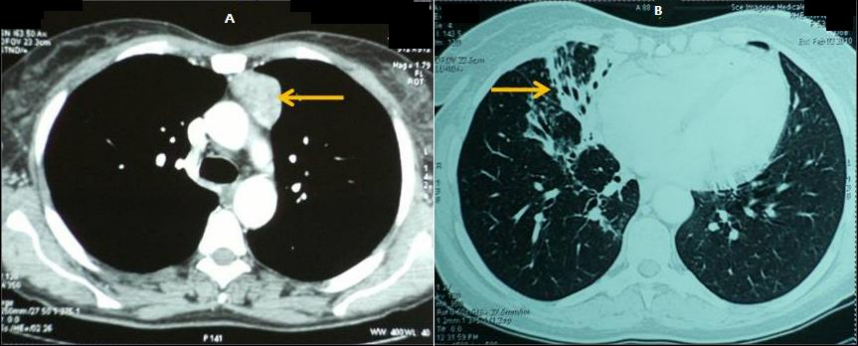
A) tomodensitométrie thoracique: masse tissulaire bien limitée du médiastin antéro-supérieur évoquant un thymome; B) tomodensitométrie thoracique: dilatations des bronches du lobe moyen et du lobe inférieur droit

Une femme âgée de 52 ans, non tabagique, ayant un diabète de type 2 a été hospitalisée pour une pneumopathie infectieuse. Cette patiente présentait depuis 2 ans des épisodes récidivants d’infections respiratoires traités en ambulatoire. L’examen physique a trouvé une fièvre à 38ºc et un foyer de râles crépitants de la base pulmonaire droite. La radiographie du thorax a mis en évidence une opacité alvéolaire du lobe moyen. La biologie a révélé un syndrome inflammatoire avec des globules blancs à 7400/mm^3^, une anémie avec une hémoglobinémie à 10,3 g/dl, une C-réactive protéine (CRP) à 48 mg/l et une vitesse de sédimentation (VS) à 50. L’examen cytobactériologique des crachats a isolé un *Streptocoque Pneumoniae*. Le diagnostic de pneumopathie bactérienne à *Streptocoque Pneumoniae* a été retenu et la patiente a été traitée par antibiothérapie adaptée. L’évolution était favorable, marquée par une défervescence thermique et une disparition du syndrome inflammatoire biologique. Sur le plan radiologique, la disparition de l’opacité alvéolaire a révélé un syndrome bronchique de la base droite. La tomodensitométrie thoracique a mis en évidence une masse tissulaire du médiastin antéro-supérieur n’envahissant pas les structures médiastinales adjacentes évoquant un thymome non invasif (Figure 1 A) ainsi que des dilatations des bronches du lobe moyen et du lobe inférieur droit (Figure 1 B). L’électromyogramme a révélé une atteinte de la jonction neuromusculaire compatible avec une myasthénie. L’électrophorèse des protéines sériques a mis en évidence une hypogammaglobulinémie avec baisse de toutes les sous-classes des immunoglobulines (Tableau 1). Le typage lymphocytaire, obtenu à partir des lymphocytes sanguins circulants, a montré une lymphopénie B, une élévation de la population lymphocytaire T CD8 et une inversion du rapport CD4/CD8. La sérologie du virus d’immunodéficience humaine (VIH) était négative. La patiente a été opérée par sternotomie et a subi une thymectomie élargie à la graisse médiastinale. L’examen anatomo-pathologique de la pièce opératoire a conclu à un thymome encapsulé de type AB de la classification de l’Organisation Mondiale de la Santé (Stade I de Masaoka). Le diagnostic de syndrome de Good associant une hypogammaglobulinémie acquise à un thymome a été retenu. Pour le traitement du déficit immunitaire humoral, la patiente a reçu des injections intraveineuses mensuelles d’immunoglobulines. L’évolution était marquée par la survenue d’infections bronchiques récidivantes.

**Tableau 1:** Electrophorèse des protides sériques montrant une hypogammaglobulinémie

	Valeurs de la patiente (g/L)	Valeurs normales (g/L)
Protides totaux	55	62-83
Albumines	33,1	36-48
Alpha 1	3,7	1-3
Alpha 2	9,8	4-8
Beta 1	4,8	4,4-6
Beta 2	2,2	1,4-3,9
Gamma	1,4	7-13

## Discussion

L’association d’un thymome à des manifestations auto-immunes est classique, retrouvée dans 74 % des cas de thymomes [2]. Il existe plus de vingt syndromes para-thymiques rapportés dans la littérature. La myasthénie en est le plus fréquent, observée chez 30% des patients [3]. L’hypogammaglobulinémie est beaucoup plus rare, ne touchant que 3 à 6% de ces patients [2,5]. Notre patiente présentait une association de deux syndromes para-thymiques, une myasthénie latente et un syndrome de Good découvert à l’occasion d’infections respiratoires basses récidivantes.

Dans la revue systématique incluant 132 patients, réalisée par Kelesidis et Yang [3], le diagnostic de thymome a précédé celui de l’hypogammaglobulinémie dans 42% des cas. L’hypogammaglobulinémie a précédé la découverte du thymome dans 20% des cas. La découverte des deux anomalies était concomitante dans près de 38% des cas. Le SG, associant thymome et déficit immunitaire, est généralement diagnostiqué au cours de la 5^ème^ ou 6^ème^ décade de la vie sans prédominance de sexe [3]. Les circonstances de découverte sont variables. Il peut s’agir d’une symptomatologie liée à la tumeur thymique associant toux, douleur thoracique, dyspnée et /ou syndrome cave supérieur [3]. La découverte fortuite d’une masse médiastinale antérieure asymptomatique est possible. Un tableau d’infections récidivantes peut enfin être inaugural, comme c’est le cas de notre patiente [4,6]. En effet, les patients ayant un syndrome de Good ont une susceptibilité accrue aux infections bactériennes, fongiques, virales et opportunistes liée à un déficit immunitaire mixte à la fois humoral et cellulaire [5,6]. Les infections les plus fréquentes sont rhino-sinusiennes et broncho-pulmonaires pouvant être à l’origine de dilatations des bronches [3-5]. Les autres infections peuvent être urinaires, osseuses ou cutanées. Les germes isolés au cours des infections respiratoires sont l’*Hemophilus influenzea* (24%), le *klebsiella* (13%) et le *Streptocoque Pneumoniae* (8 à 13%) [2,4,5]. Le *Pseudomonas Aeruginosa* a été également isolé chez les patients ayant des dilatations des bronches. Le SG est souvent associé à de nombreuses manifestations hématologiques. Ainsi, l’anémie est présente chez 50% à 86% des patients [3,6]. Les autres manifestations regroupent l’érythroblastopénie, l’anémie hémolytique et les syndromes myélodysplasiques. La leucopénie est retrouvée chez 46% à 55% des cas [3,4,6].

La relation entre le thymome et l’hypogammaglobulinémie reste mal connue. Sur le plan immunologique, les principales anomalies retrouvées au cours de ce syndrome sont une hypogammaglobulinémie, une absence de lymphocytes B circulants, un rapport CD4/CD8 anormal avec une expansion de la sous population lymphocytaire T CD8 et une absence de réponse des lymphocytes T aux mitogènes [3,6,7]. L’absence de lymphocytes B semble secondaire à un blocage très précoce de la différenciation B au niveau médullaire [4,7,8]. Au cours du SG, une hypogammaglobulinémie touchant toutes les sous classes d’immunoglobulines était notée dans 75% des cas [3]. Plus rarement, une diminution isolée des IgG et des IgM était rapportée dans 9,1% et 1,8% respectivement [3].

La découverte à l’électrophorèse des protéines sériques d’une hypogammaglobulinémie doit conduire à la réalisation d’une exploration immuno-hématologique à la recherche d’une étiologie. En effet, de nombreuses pathologies peuvent être à l’origine d’une hypogammaglobulinémie et doivent être éliminées avant de retenir le diagnostic de SG. Il s’agit principalement de la leucémie lymphoïde chronique, l’infection par le HIV, le lymphome et le myélome multiple [3-5]. De même, certains médicaments comme les antiépileptiques et les immunosuppresseurs peuvent engendrer une hypogammaglobulinémie [3-5]. Ces médicaments doivent être recherchés par une anamnèse minutieuse.

Le traitement repose sur la résection chirurgicale de la tumeur thymique, qui reste sans efficacité évidente sur les manifestations auto-immunes. En effet, même après thymomectomie, il persiste une susceptibilité aux infections liée à une hypogammaglobulinémie durable [7,8]. Ainsi, les immunoglobulines intraveineuses peuvent être indiquées pour mieux contrôler les infections souvent sévères pouvant mettre en jeu le pronostic vital [9]. Le pronostic du syndrome de Good semble péjoratif avec une survie respective à cinq et dix ans de 70 % et 30 % [5,6]. Chez notre patiente, comme rapporté dans la littérature [7,8], la thymomectomie était inefficace sur l’hypogammaglobulinémie motivant un traitement substitutif par injections intraveineuses d’immunoglobulines n’ayant pas prévenu la survenue de complications infectieuses.

## Conclusion

Le syndrome de Good, bien que rare, peut constituer l’étiologie des dilatations des bronches. L’électrophorèse des protéines sériques est un examen simple qui doit être réalisé chez les patients ayant des infections respiratoires basses récidivantes et/ou des dilatations des bronches particulièrement si un thymome est associé. La découverte d’une hypogammaglobulinémie profonde doit conduire à la réalisation d’un bilan immuno-hématologique qui permettra d’éliminer une hémopathie lymphoïde B et préciser le type du déficit immunitaire acquis.

## Conflits d’intérêts

Les auteurs ne déclarent aucun conflit d’intérêts.
